# Numerical Method for the Design of Healing Chamber in Additive-Manufactured Dental Implants

**DOI:** 10.1155/2017/1970680

**Published:** 2017-02-12

**Authors:** Hsiao-Chien Lee, Pei-I Tsai, Chih-Chieh Huang, San-Yuan Chen, Chuen-Guang Chao, Nien-Ti Tsou

**Affiliations:** ^1^Department of Materials Science and Engineering, National Chiao Tung University, Hsinchu 30010, Taiwan; ^2^Biomedical Technology and Device Research Laboratories, Industrial Technology Research Institute, Chutung, Hsinchu 31040, Taiwan

## Abstract

The inclusion of a healing chamber in dental implants has been shown to promote biological healing. In this paper, a novel numerical approach to the design of the healing chamber for additive-manufactured dental implants is proposed. This study developed an algorithm for the modeling of bone growth and employed finite element method in ANSYS to facilitate the design of healing chambers with a highly complex configuration. The model was then applied to the design of dental implants for insertion into the posterior maxillary bones. Two types of ITI® solid cylindrical screwed implant with extra rectangular-shaped healing chamber as an initial design are adopted, with which to evaluate the proposed system. This resulted in several configurations for the healing chamber, which were then evaluated based on the corresponding volume fraction of healthy surrounding bone. The best of these implants resulted in a healing chamber surrounded by around 9.2% more healthy bone than that obtained from the original design. The optimal design increased the contact area between the bone and implant by around 52.9%, which is expected to have a significant effect on osseointegration. The proposed approach is highly efficient which typically completes the optimization of each implant within 3–5 days on an ordinary personal computer. It is also sufficiently general to permit extension to various loading conditions.

## 1. Introduction

Dental implant has been an important surgical component in recent years. A well-designed dental implant is able to benefit patients with improved appearance, comfort, and speech, as well as stopping the bone loss [1, 2] and enhancing the structural connection between bone and the surface of the implant, that is, the osseointegration [3, 4]. In the recent decades, the concept of a healing chamber has been introduced to the design of threaded dental implants [5, 6]. The healing chamber is defined as the voids between adjacent threads, where the implant and the bone are not initially in contact right after the implant placement [7]. The voids are then filled with blood clots, which can further form the osteogenic tissue leading to the ingrowth of woven bone [6, 8]. The chamber significantly alters the biological healing pattern, compared to it in the case of the traditional screw root shape implants [8, 9].

Most studies on the design of healing chambers have focused on the shape of the threads, the thread pitch [1, 2, 10, 11], and the dimensions of surgical drilling [3, 7, 12, 13]. Beutel et al. [14] determined that an implant with a trapezoidal healing chamber is best for osseointegration, while those with the upper triangle shaped chambers delay the bone ingrowth. Coelho et al. [15] studied the effect of the size of the chamber and the implant surface treatments and concluded that the small chamber presents a better biomechanical fixation in the cases they considered. Marin et al. [7] also suggested that the depth and height of the healing chamber should be limited. This is because the blood clot may not fully fill in the oversized healing chamber, resulting in a poor osseointegration. Lossdörfer et al. [16] and dos Santos et al. [17] reported that acid etched implants provide surface roughness, greater surface area, and better stability compared with the machined implants.

Circumferential troughs within the region between threads can also serve as a healing chamber. In an in vivo study, Berglundh et al. [5] demonstrated the formation of bone and resulting stability of implants with U-shaped circumferential troughs. Buser et al. [18] and Abrahamsson et al. [19] also adopted the implants with the similar design and studied the effect of surface treatment on the bone apposition to the surface. The advantage of the implant of this type is that the primary mechanical stability of the implant can be preserved by the threads with a smaller surgical drilling dimension, and the secondary stability can still be established by the osseointegration in the troughs [5].

The design of the healing chamber is also greatly affected by the manufacturing techniques. Dental implants are typically fabricated using Ti6Al4V. The complexity of the implant shape is limited by machining techniques [22]. The machining procedure is quite challenging and costly specifically when the implant is required to be in a complicated or customized configuration [23, 24]. Thus, the configuration of the conventional healing chambers is limited to be in simple shapes, such as triangle, rectangle, and trapezoid [14]. The recent introduction of additive manufacturing to the fabrication of implants has made it possible to create designs of far greater complexity [22]. For example, Traini et al. [25] adopted a direct laser metal sintering technique to prepare porous titanium dental implants, which have a better elastic adaptability to the bone, minimizing the stress shielding effects. Stübinger et al. [26] used a similar technique to manufacture a dental implant with complicated gyroid geometry, forming repeated pores on the implant. It was applied to the case of the patients with compromised bone situations, resulting in sound osseointegration. However, most of the design guidelines pertaining to the healing chamber are based on specific loading conditions or parametric studies inapplicable to the design of additive-manufactured implants. Adopting a systematic approach to the design of healing chambers applicable to any loading conditions could be highly beneficial.

Extending the applicability of design methods to include cases with a wider range of loading conditions would require that researchers to take into account the remodeling of bone after implantation. The mechanism underlying bone remodeling is described by Wolff's Law [27] and the Mechanostat hypothesis [28]. The central idea of the theories is that, when the bone is subjected to an adequate level of strain, the bone will strengthen itself to resist the load caused by the strain and, when the strain decreases to the certain levels, osteoclasts will absorb the bone tissue. This has been extensively applied to the simulation of the bone remodeling and analysis of the implant design. Hasan et al. [29] developed a finite element model of a screw-shaped dental implant. The model updates the bone density iteratively to simulate the process of the bone remodeling. Chou et al. [30] and Lin et al. [31] adopted the adaptive strain energy density algorithm to predict the variation of the bone density around implants under different loads. Kwon et al. [32], Adachi et al. [33], and Tsubota et al. [34] also developed models capable of simulating bone growth through the addition and removal of voxel elements. This element-based approach is particularly suitable for the application to the design of additive-manufactured implants and, thus, is adopted in the current study.

In this work, which is extended by the authors' previous model [35], a numerical method by which a computer program is used to “grow” the configuration of a healing chamber is proposed, based on the modified finite element approach proposed by Kwon et al. [32]. As the implant is subjected to given loads, based on Wolff's Law, certain regions of the bone element in the healing chamber may be removed when the strain value in these regions is lower than a given threshold. This indicates that the bone cells in these regions are less likely to survive under the given loads. Thus, in the current method, the implant elements are then filled into the regions which are originally occupied by the removed elements of the bone, in order to maximize the implant surface area. Similarly, when the bones in the certain regions of the healing chamber attempt to increase the strength to resist the loads by expanding the volume of the region (adding bone elements), the implant elements in the corresponding region will be replaced by the newly formed bone elements. This iterative procedure is analog to the bone remodeling with the difference that the configuration of the healing chamber is varying at each iteration. The implant geometry at each iteration can then be a candidate for the best implant design for the given loads.

While conventional dental implants typically adopt the same shape of healing chambers throughout the entire implant, the modified finite element approach can generate a biomimetic dental implant which allows the diversity of the shape of the healing chamber across different regions between threads. This complex shape of the healing chamber is the result of the complicated local boundary conditions and is particularly suitable for the additive manufacturing. In the following sections, the algorithm of the bone remodeling and the corresponding 3D finite element model will be described. The power of this method with detailed study of the dental implants used in posterior maxillary bones is then illustrated. The approach is sufficiently general to permit extension to other types of bone as well as various of loading conditions.

## 2. Theory and Methodology

### 2.1. Algorithm of Bone Remodeling

The proposed numerical model is based on the methods outlined by Kwon et al. [32, 36], wherein the structure of the bone is discretized. The addition and removal of bone elements are determined by the value of local equivalent strain *ε*. It is assumed that a decrease in the equivalent strain accelerates bone resorption when *ε* < *ε*_*du*_, such that the formation of bone would occur at an accelerated rate following an increase in *ε* when *ε*_*ol*_ ≤ *ε*. The two strain ranges are known as the disuse window (DW) and overuse window (OW), respectively. When *ε*_*pl*_ ≤ *ε* < *ε*_*pu*_, the strain range is referred to as a physiological window (PW), in which bone formation and resorption are assumed to occur stochastically according to the degree of nonuniformity in local stress.

Now consider point *c* on the bone surface, where the equivalent strain of the corresponding bone element is *ε*_*c*_. The probability of adding or removing a new bone element at point *c* which is denoted by *f*^*∗*^(*Г*_*c*_, *ε*_*c*_) is written as follows [32]:

(1) $$\begin{array}{c} f^{*}\left(\;{Г}_{c},\varepsilon_{c}\;\right)\left\{\begin{array}{cc} -1, & \varepsilon_{c}<\varepsilon_{du},DW,\\ -\left(1-\frac{\varepsilon_{c}-\varepsilon_{du}}{\varepsilon_{pl}-\varepsilon_{du}}\right)+\left(\frac{\varepsilon_{c}-\varepsilon_{du}}{\varepsilon_{pl}-\varepsilon_{du}}\right)f\left({Г}_{c}\right), & \varepsilon_{du}\leq \varepsilon_{c}<\varepsilon_{pl},\\ f\left({Г}_{c}\right), & \varepsilon_{pl}\leq \varepsilon_{c}<\varepsilon_{pu},PW,\\ \left(1-\frac{\varepsilon_{c}-\varepsilon_{pu}}{\varepsilon_{ol}-\varepsilon_{pu}}\right)f\left({Г}_{c}\right)+\left(\frac{\varepsilon_{c}-\varepsilon_{pu}}{\varepsilon_{ol}-\varepsilon_{pu}}\right), & \varepsilon_{pu}\leq \varepsilon_{c}<\varepsilon_{ol},\\ 1, & \varepsilon_{ol}\leq \varepsilon_{c},OW, \end{array}\right. \end{array}$$

where *f*(*Г*_*c*_) is the probability of bone resorption and formation at point* c*, which is a function of nonuniformity (*Г*_*c*_) in the stress distribution in the bone near point* c*.

### 2.2. Numerical Model for the Design of Healing Chambers

**Figure 1:**
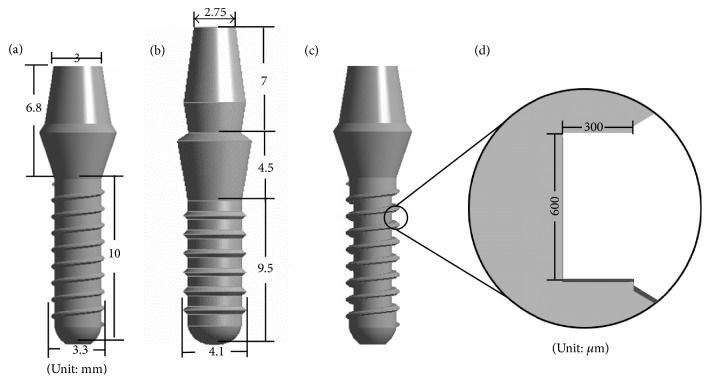
Geometry of (a) type (1), (b) type (2) of ITI commercial implants, (c) type (1) implant with healing chamber as an initial implant, and (d) the cross-section of the healing chamber of initial implant.

In the following, the algorithm used to design the trough-type healing chamber in dental implants is outlined. The cross-section of the healing chamber is initially set as a simple rectangle, as illustrated in Figure 1(d). Here, it is assumed that the bone elements occupy the entire region of the healing chamber in every iteration. Contact and target surface elements are applied to the interface at every point at which the bone and implant elements meet. For the sake of computational efficiency, linear contact mode is applied, and bonded contact conditions are set at the interface.

The implant is then subjected to specific loads and boundary conditions. The equivalent stress and strain states of each bone element in contact with implant elements at the surface of the healing chamber are substituted into Equation (1) to determine the probability of bone elements being added or removed. In the case where a bone element is removed, the region of removal is treated as an area in which bone tissue will be absorbed under the current boundary conditions, thereby leaving an empty space. According to the literature [37], osseointegration can be improved by increasing the bone-implant contact surface area. Thus, any empty spaces are filled with an implant element. However, in the case where bone elements are added, an implant element in contact with a bone element is replaced by a new bone element. This process of replacement may be repeated several times, depending on the activation frequency [36]. This makes it possible to simulate the rate dependence of bone remodeling.

It is natural that the bone elements within a healing chamber be subjected to stress and strain conditions lower than those outside the healing chamber. The mechanism of nonuniformity *Г*_*c*_ tends to remove bone elements from inside the healing chamber to achieve a more uniform stress state in the bone tissue. This makes it inevitable that after several iterations the healing chamber will be entirely filled with implant elements. Thus, the configuration of the healing chamber in its final iteration is not necessarily the best implant design for the given load. Thus, the configurations generated in every iteration are regarded as candidates to be evaluated according to the volume fraction of the healthy surrounding bone in the region of interest. Healthy bone is defined as bone tissue under a strain state similar to that caused by normal physical activities, ranging between 400 *με* and 1500 *με* [28]. The candidate with the greatest volume fraction of healthy surrounding bone is regarded as the implant with the best healing chamber.

## 3. Application to Dental Implants in Posterior Maxillary Bones

### 3.1. Establishment of Three-Dimensional Model

In the following, the proposed numerical method is applied to the design of implants with healing chamber for implantation in the posterior maxillary bone. Two types of implant are studied here: (1) a commercial ITI (Institute Straumann AG, Waldenburg, Switzerland) solid cylindrical screwed implant number 033.512S [21] and (2) another commercial ITI implant number 033.563S [31] as shown in Figures 1(a) and 1(b), respectively.

**Figure 2:**
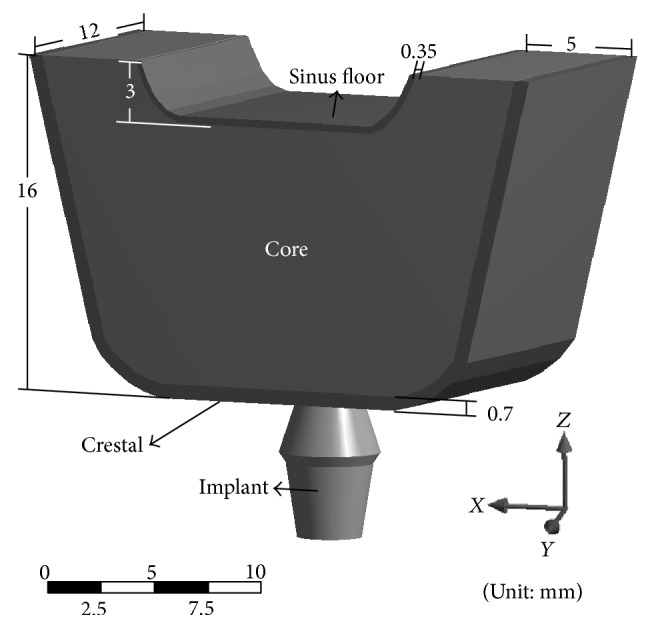
Three-dimensional model showing dental implant and maxillary posterior bone structure.

The initial configuration of both types of implant was based on the design of the ITI devices with an added circumferential trough. The depth of the trough is chosen to be the half of spacing between pitches. Figure 1(c) shows type (1) implant with the added circumferential trough, and the cross-section of the trough presents a simple rectangle as shown in Figure 1(d).  Figure 2 illustrates the overall bone structure, which was adopted from the model developed by Li et al. [21].

**Figure 3:**
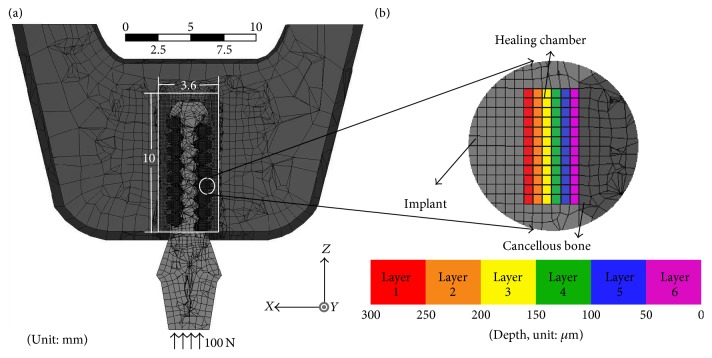
(a) Cross-section view of meshed implant and bone structure with applied boundary conditions. The cylindrical region marked by white solid lines is the region used in the evaluation of the volume fraction of healthy surrounding bone. (b) Details of the elements in the design region. The color legend indicates the depth of the element from the deepest layer (layer 1) to the surface of the implant (layer 6) in the case of type (1) implant.

The corresponding finite element model was constructed and meshed using ANSYS Workbench 15.0. The element type SOLID 185 was used for all implant and bone elements. The model for the case of type (1) implant is shown in Figure 3(a) for illustration purpose. A circumferential trough was stipulated as the region used for the design of the healing chamber; that is, implant and bone elements can only be added or removed within that region. The device was meshed using the sweep method for the generation of regular 8-node-hexahedron elements with a width of 50 *μ*m, which corresponds to the size of the bone elements used in previous bone models [38]. This is similar to the average particle size of Ti6Al4V in additive manufacturing [39]. Figure 3(b) illustrates the elements in the layers, wherein different colors are used to indicate the depth of the healing chamber. Note that the cylindrical region marked by solid white lines in Figure 3(a) is the region used to evaluate the volume fraction of healthy surrounding bone for the case of type (1) implant.

The parameters used in the model follow those outlined by Kwon et al. [32].  Table 1 lists the mechanical properties of the bone and implant, where type IV bone is considered in the current model for modeling the composition in posterior jaw. The displacement components of nodes on the surface of the mesial and distal bone regions (in the positive and negative Y-direction) are constrained. The loading conditions for type (1) implant are motivated by Li et al. [21], where 100 N compressive uniaxial loading is uniformly applied along the positive *Z*-axis on the top surface of the abutment, as shown in Figure 3(a), while the magnitude of the loading for type (2) implant is 175 N, motivated by Lin et al. [31].

### 3.2. Results

#### 3.2.1. The Candidates of the Implant Generated by the Current Algorithm

**Figure 4:**
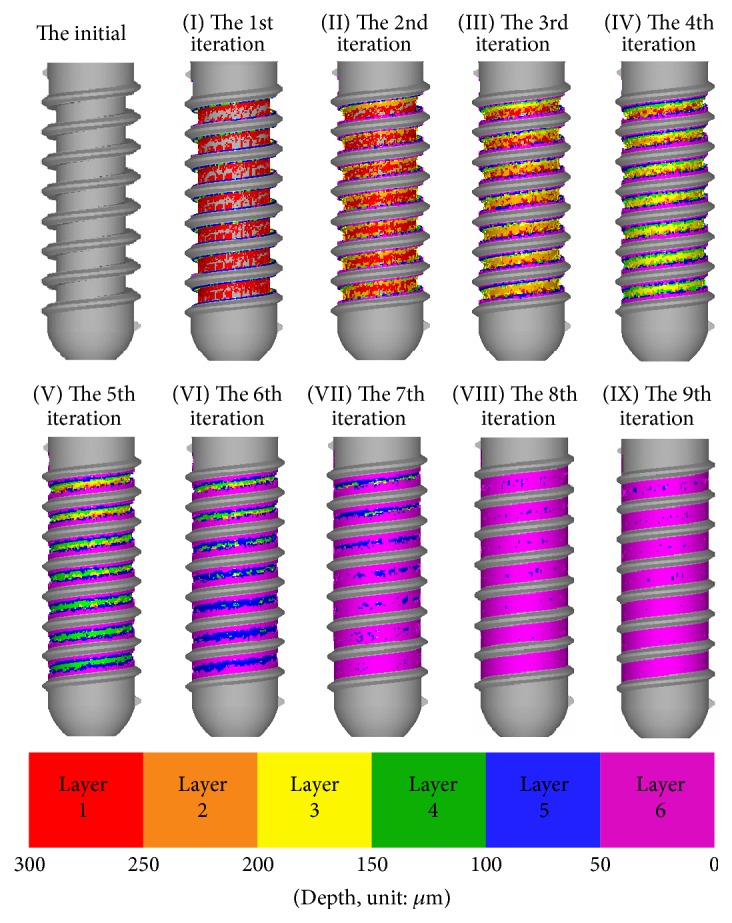
Geometry of initial implant and implants I–IX generated in each iteration of type (1) implant.

Now consider type (1) implant (ITI number 033.512S).  Figure 4 illustrates the changes on the shape of the area between threads of the initial implants and implants I–IX generated in each iteration of the design process for type (1) implant. Note that implant elements are assigned using distinct colors (red-purple) to indicate their depth.  Figure 4(I) presents the implant generated in the 1st iteration of the design process, showing the large number of implant elements added to layer 1 (red) of the healing chamber. These implant elements form a pattern of square shapes and bands beneath the threads and will be discussed in more detail in Section 3.2.3. As shown in Figure 4(IV), in the 4th iteration, the depth of the healing chamber decreased. Several implant elements were added along both sides of the threads, which resulted in an eagle's beak configuration. In samples generated beyond the 4th iteration, the depth and height of the healing chamber gradually decrease and the eagle's beak configuration vanishes as the bone remodeling mechanism seeks a uniform stress state. By the 9th iteration, the entire design region is filled with implant elements.

**Figure 5:**
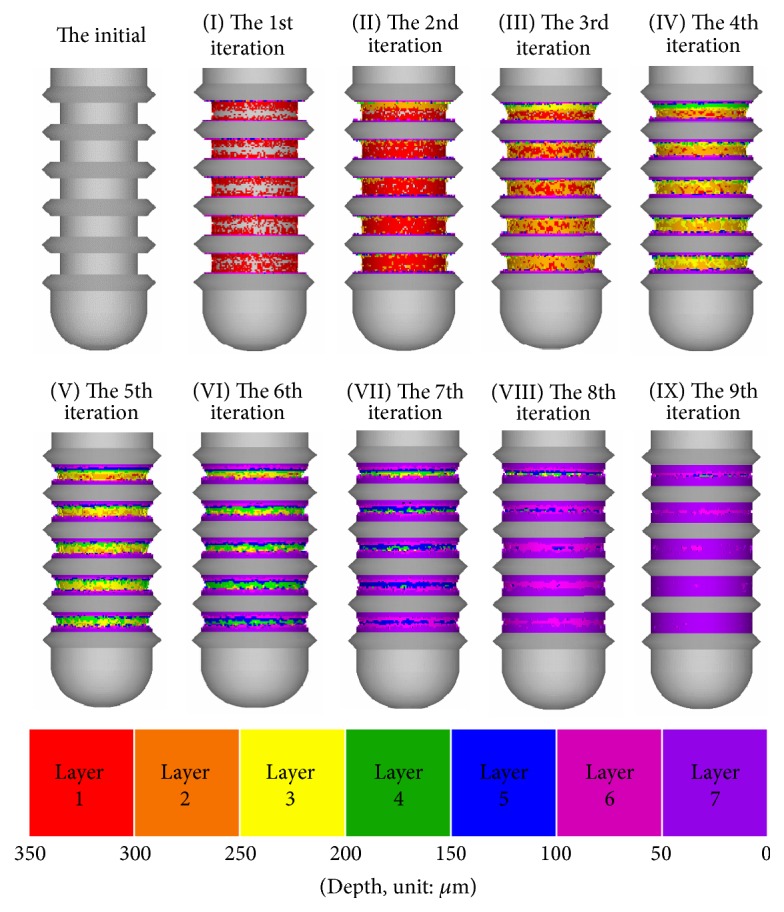
Geometry of initial implant and implants I–IX generated in each iteration of type (2) implant.

Now consider type (2) implant (ITI number 033.563S). The changes on the shape of the area between threads of the initial implant and implants I–IX generated in each iteration of the design process for type (2) implant are illustrated in Figure 5. Note that the depth of the healing chamber in type (2) implant is 350 *μ*m, and thus seven distinct colors (red-deep purple) are used here to indicate their depth. Figure 5(I) shows that the large number of implant elements is added as bands above and beneath the threads, forming small troughs in the healing chamber. Similar to the case of type (1), the depth and height of the healing chamber gradually decrease as the bone remodeling mechanism seeks a uniform stress state, and the entire design region in the 9th iteration is almost filled with implant elements.

#### 3.2.2. The Volume Fraction of Healthy Surrounding Bone

**Figure 6:**
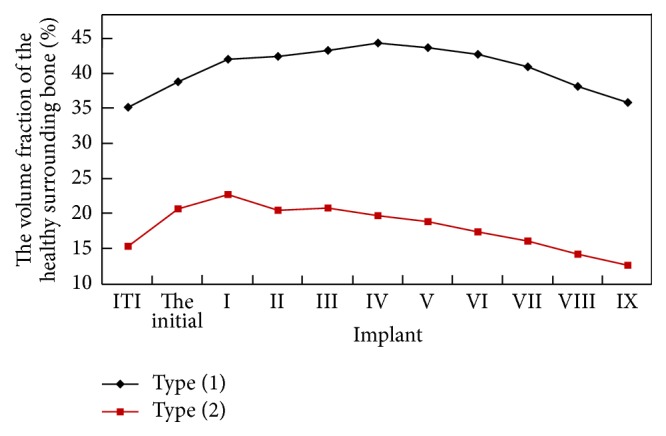
Volume fraction of healthy surrounding bone corresponding to two types of ITI commercial implant along with the initial implants and implants I–IX generated from each iteration.

The volume fraction values of healthy surrounding bone for both types (1) and (2) implants are presented in Figure 6. Due to the new design of the shape between threads, all of the implants with a trough-type healing chamber exceeded their original design of the ITI commercial implants with regard to the volume fraction of healthy surrounding bone, which demonstrates the efficacy of this approach to the remodeling of bone. In the case of type (1), shown as a black line with diamond markers in Figure 6, implant IV presents the largest volume fraction of healthy surrounding bone (44.4%). The total contact area between the implant and bones of implant IV is 167.8 mm^2^, which exceeds type (1) ITI commercial implant by almost 53%.

Consider the case of type (2). The volume fractions of healthy surrounding bone for all the implants, illustrated as a red line with square markers in Figure 6, are below those in the case of type (1) due to the effect of the design of the geometry and the applied loads. The data shows that implant I results in 22.8% healthy surrounding bone which is the greatest value among the other candidates of the implant generated by the algorithm. The total contact area between the implant and bones of implant I is 214.5 mm^2^, which exceeds type (2) ITI commercial implant by about 42%. The improvements for both types (1) and (2) implants would undoubtedly enhance the osseointegration [41, 42], which would help to prevent damage to the bone [43]. Thus, implant IV in case (1) and implant I in case (2) are regarded as the implants with the best healing chamber under the given loads.

#### 3.2.3. The Strain Contour of the Surrounding Bone

**Figure 7:**
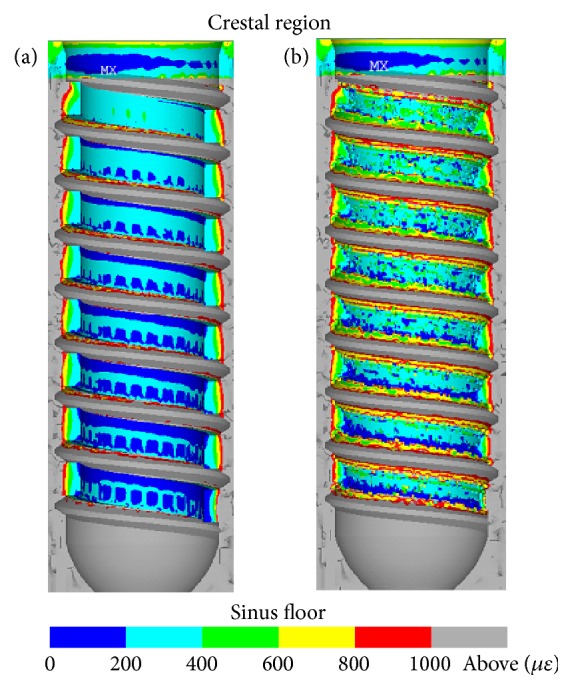
Strain contour in a cross-section of the bone surrounding healing chamber of (a) initial implant and (b) implant IV in the case of type (1).

Next, the strain state of the surrounding bone for both cases (1) and (2) is examined in order to reveal the effect of the design of the healing chambers.  Figure 7(a) illustrates the equivalent strain contour of the bone surrounding the initial implant in the case of type (1). Most of the bone elements in the design region close to the sinus floor are at strain states below 200 *με*. The strain contour of these elements forms a pattern of square shapes and bands beneath the threads, which shrink toward the crestal region. This is an indication that bone elements close to the crestal region are in higher strain states. Nonetheless, the strain state of bone elements within the healing chamber remains low, in terms of the bone remodeling criteria. This can also be seen in the second data point of the black line in Figure 6, where the healthy surrounding bone accounts for a volume fraction of only 38% in the area of concern. This can explain why implant I generated in the 1st iteration gives a pattern similar to that of the low-strain bone elements used in the initial calculation, which indicates that the low-strain bone elements are more likely to be absorbed and, thus, replaced by implant elements in the proposed algorithm.  Figure 7(b) presents the strain contour of the bone surrounding the healing chamber of implant IV, in which the pattern of square shapes vanishes and the strain state of the bone elements becomes more uniform. Furthermore, the region of low-strain bone elements is reduced to a thin band near the sinus floor as higher strain state bone elements are introduced to the healing chamber.

**Figure 8:**
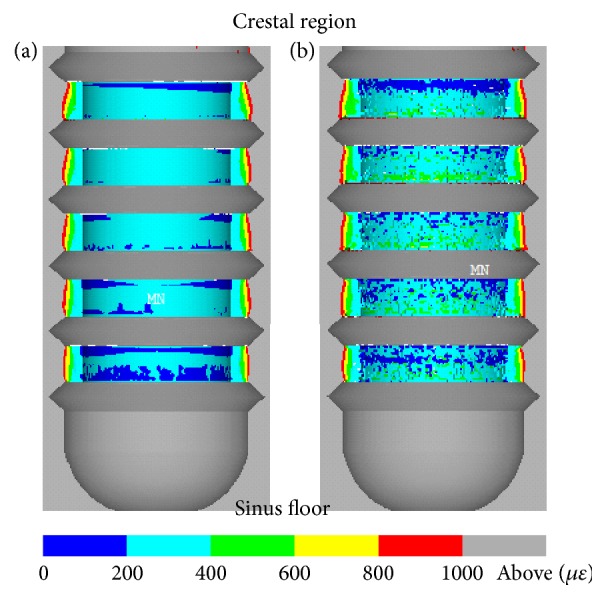
Strain contour in a cross-section of the bone surrounding healing chamber of (a) initial implant and (b) implant I in the case of type (2).

Now consider the case of type (2).  Figure 8(a) shows the equivalent strain contour of the bone surrounding the initial implant. Several bands of strain at around 200 *με* can be found above and beneath the threads close to the sinus floor. Most of the bone elements in the design region of the initial implant are at strain states in the range of 200 *με* and 400 *με*. In the next iteration, the resulting implant I gives the strain contour as shown in Figure 8(b). It can be observed that the strain state in the region above each thread reaches the range of 400 *με* and 600 *με*, where the bone is in a healthy status and is less likely to be absorbed.

### 3.3. Discussion

**Figure 9:**
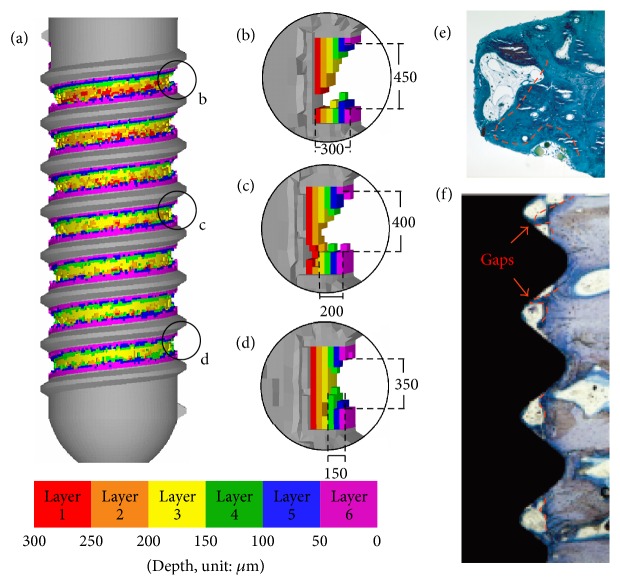
(a) Implant IV in the case of type (1) and details of configuration of healing chamber at (b) top, (c) middle, and (d) bottom of implant. (e), (f) The outline of the formation of bone illustrated by the dashed red line (Berglundh et al. [5, 40]).

**Figure 10:**
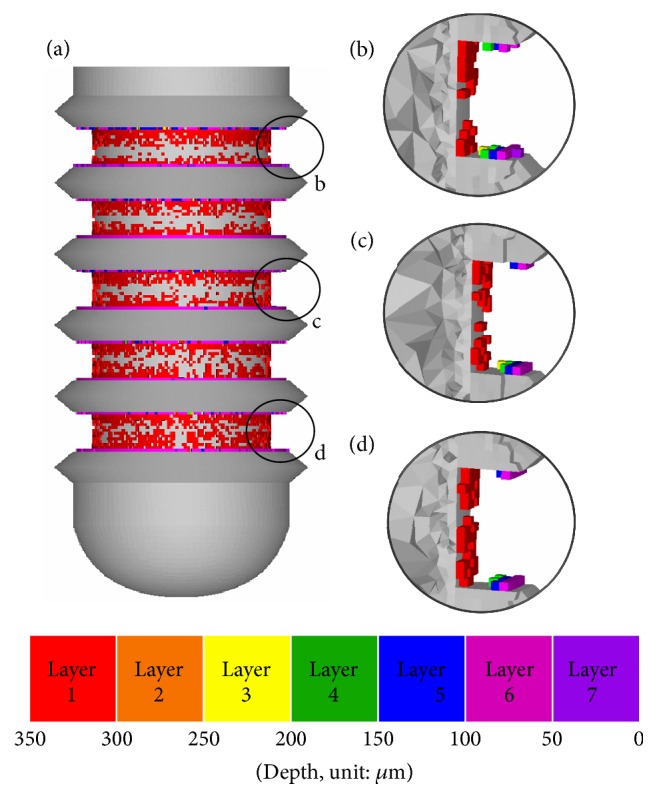
(a) Implant IV in the case of type (2) and details of configuration of healing chamber at (b) top, (c) middle, and (d) bottom of implant.

Based on the results reported in the previous section, the design generated by the current algorithm can increase the volume fraction of the healthy surrounding bone and the bone-implant contact area. The design shares several features with those reported in the past works.  Figure 9(a) presents a cross-section of implant IV of case (1) showing details of the top, middle, and bottom areas of the healing chamber between threads (Figures 9(b), 9(c), and 9(d), respectively). The healing chamber of implant IV shares several features seen in the optimized dental implants reported by previous researchers. The cross-section of the top of the healing chamber appears in the shape of an eagle's beak which is in good agreement with the in vivo remodeling results reported by Berglundh et al. [5].  Figure 9(e), courtesy of Berglundh et al. [5], shows that the remodeled bone formed low-strength marrow regions located at the lower front and the entire back surface of the U-shaped healing chamber, which is also similar in appearance to an eagle's beak (illustrated by the dashed red line). As shown in Figure 9(c), the depth of the middle area of the healing chamber is approximately 200 *μ*m. This decrease in depth can be attributed to the fact that bone elements in the middle of the implant are subjected to less strain than those close to the crestal region. The bottom of the healing chamber tapers down to a depth of 150 *μ*m with a height of 350 *μ*m in a trapezoidal configuration (Figure 9(d)), which was reported as advantageous by Beutel et al. [14]. Marin et al. [7] recommended a small healing chamber for bone remodeling. The tapering from the top to the bottom of the healing chamber is in agreement with the results obtained in in vivo studies of bone growth, as shown in Figure 9(f) (courtesy of Berglundh et al. [40]). The outline of the formation of bone is illustrated by the dashed red line, where larger quantities of bone formed an eagle's beak shape in the healing chamber close to the crestal region, while less formed a trapezoidal shape in the area close to the sinus floor.  Figure 10 shows the detail of the optimized implant I of case (2). The cross-section of the top, middle, and bottom areas of the healing chamber between threads appear to be similar. A feature in common is that there is a gap in layer 1 (red color) in the healing chambers, which is also in good agreement with the gaps found in Figure 9(f) [40].

The proposed algorithm redesigns the geometry between threads of the two types of commercial implants, giving improved volume fraction of healthy surrounding bone and surface and implant contact area. The optimized healing chamber in both cases varies across the entire body of the implant according to local boundary conditions giving good agreement with in vivo remodeling results reported in the literature. Additive manufacturing is ideally suited to such a design.

## 4. Conclusions

This paper presents a novel numerical model for the automatic configuration of healing chambers based on a given load. An algorithm based on this bone remodeling mechanism is capable of creating biomimetic implants ideally suited to additive manufacturing. The proposed model was applied to the design of a trough-type healing chamber on two types of ITI dental implant for insertion into the posterior maxillary bones. The resulting healing chamber includes several of the design features recommended by researchers, such as trapezoidal and eagle-beak shapes. Compared to the original commercial implant, these designs increase the volume fraction of the healthy surrounding bone and the total contact area between the implant and bones for both cases. These improvements are expected to promote osseointegration in the cases considered here. Although the current paper proposes a pure theoretical work which may not be directly translated into clinical reality, due to several different factors related to healing or the accuracy of 3D printer, the proposed approach still provides several design guidelines. It is highly efficient (3–5 days on an ordinary personal computer for one case) and sufficiently general to enable application to any type of implant, for any type of bone structure, under any load conditions. This is an ideal tool for the customization of dental implants and improvement of the design process.

## Figures and Tables

**Table 1:** Mechanical properties of materials used in simulation.

Materials	Young's modulus (GPa)	Poisson ratio	Reference
Cortical bone	13.7	0.3	[20]
Cancellous bone	0.69	0.3	[21]
Ti6Al4V	110.0	0.35	[20]
